# Using Musculoskeletal Models to Estimate *in vivo* Total Knee Replacement Kinematics and Loads: Effect of Differences Between Models

**DOI:** 10.3389/fbioe.2021.703508

**Published:** 2021-07-28

**Authors:** Cristina Curreli, Francesca Di Puccio, Giorgio Davico, Luca Modenese, Marco Viceconti

**Affiliations:** ^1^Department of Industrial Engineering, Alma Mater Studiorum - University of Bologna, Bologna, Italy; ^2^Medical Technology Lab, IRCCS Istituto Ortopedico Rizzoli, Bologna, Italy; ^3^Dipartimento di Ingegneria Civile e Industriale, Università di Pisa, Pisa, Italy; ^4^Department of Civil and Environmental Engineering, Imperial College London, London, United Kingdom

**Keywords:** musculoskeletal modeling, joint contact forces, knee implant kinematics, total knee replacement, model credibility assessment

## Abstract

Total knee replacement (TKR) is one of the most performed orthopedic surgeries to treat knee joint diseases in the elderly population. Although the survivorship of knee implants may extend beyond two decades, the poor outcome rate remains considerable. A recent computational approach used to better understand failure modes and improve TKR outcomes is based on the combination of musculoskeletal (MSK) and finite element models. This combined multiscale modeling approach is a promising strategy in the field of computational biomechanics; however, some critical aspects need to be investigated. In particular, the identification and quantification of the uncertainties related to the boundary conditions used as inputs to the finite element model due to a different definition of the MSK model are crucial. Therefore, the aim of this study is to investigate this problem, which is relevant for the model credibility assessment process. Three different generic MSK models available in the OpenSim platform were used to simulate gait, based on the experimental data from the fifth edition of the “Grand Challenge Competitions to Predict *in vivo* Knee Loads.” The outputs of the MSK analyses were compared in terms of relative kinematics of the knee implant components and joint reaction (JR) forces and moments acting on the tibial insert. Additionally, the estimated knee JRs were compared with those measured by the instrumented knee implant so that the “global goodness of fit” was quantified for each model. Our results indicated that the different kinematic definitions of the knee joint and the muscle model implemented in the different MSK models influenced both the motion and the load history of the artificial joint. This study demonstrates the importance of examining the influence of the model assumptions on the output results and represents the first step for future studies that will investigate how the uncertainties in the MSK models propagate on disease-specific finite element model results.

## Introduction

Total knee replacement (TKR) surgeries are commonly performed to alleviate severe pain at the knee joint resulting from musculoskeletal (MSK) disorders/conditions (e.g., inflammatory arthritis) that mostly affect elderly patients. In recent times, this is considered an effective procedure in orthopedics with a failure rate at 10 years postoperatively lower than 5% (Khan et al., 2016). But because of the popularity of this surgical procedure in the aging population, even 5% of failures produce some 10,000 revision surgeries every year in Europe. In contrast, patient-reported outcome measures suggest that 20–30% of the patients are not happy with the functional outcome of the surgery (Nakano et al., 2020). This calls for the development of new better-performing designs, whose safety and efficacy could be conveniently evaluated at the very early phases of the design taking advantage of the growing use of numerical modeling techniques.

A promising modeling strategy in the field of computational biomechanics that can be used to accelerate implant design innovation and improve subject-specific TKR outcomes is based on the combination of MSK multibody-dynamics analysis and finite element analysis (FEA) (Zhang et al., 2017; Shu et al., 2020b). The MSK modeling technique uses the classic multibody approach to estimate rigid body mechanics (i.e., joint kinematics and dynamics with ideal joint actuators) combined with muscle models to estimate muscle forces (i.e., replacing actuators with muscles) and joint reactions (JRs) by means of numerical optimization techniques. Additionally, FEA is a powerful tool to predict tissue-level mechanics such as contact pressure and surface damage of the implant surfaces. The main idea is that, by using the outputs of the MSK simulations in terms of load history and motion of the artificial joint as input conditions for the finite element model, it is possible to obtain a more realistic prediction of the joint biomechanics for each patient. This approach has been recently used in the literature to address specific research questions. Zhang et al. (2017) developed a combined MSK–FEA patient-specific computational wear prediction framework that estimates the damaging process in the tibial insert (TI) during gait in a patient implanted with an instrumented prosthesis. An *in silico* wear simulator was also developed by Shu et al. (2020a) using finite element models and loading conditions of different daily activities obtained by MSK modeling. Recently, a multiscale forward-dynamic framework of the lower extremity that combined muscle modeling and deformable FEA was presented in the study by Hume et al. (2019). This approach was used to predict healthy joint mechanics during different physical activities, and it is considered a promising strategy for the preclinical evaluation and design of TKR.

One of the most crucial aspects of this combined modeling approach is the definition of the MSK model that best describes both the anatomy and the physiology of the specific patient. Subject-specific MSK models that use medical images to create individualized geometries and properties of the patient under investigation are, in recent times, an attractive solution for the problem (Marra et al., 2015; Valente et al., 2017; Modenese et al., 2018). However, they require complex modeling workflows and *ad hoc* data collection even when applying well-documented approaches (Modenese et al., 2018), so the use of generic MSK models is in most cases the preferred solution due to its feasibility, at least until fully automated approaches become available (Modenese and Kohout, 2020; Modenese and Renault, 2021). Generic models are based on cadaveric data and mainly differ from each other in the MSK anatomy, kinematic of the joints and coordinate system definition, degrees of freedom, number, and properties of the muscles, and other biological structures such as ligaments. Numerous generic MSK models with varying levels of complexity have been presented in the literature over the last years (Delp et al., 1990; Anderson and Pandy, 1999; Arnold et al., 2010; Modenese et al., 2011; Marra et al., 2015; Rajagopal et al., 2016; Lai et al., 2017), giving a wide range of possible choices for this study. Thus, selecting the most suitable model for a specific study is challenging and requires a detailed understanding of the effect of different model parameters on the simulation results. Recent studies tried to address this problem by investigating the sensitivity of the estimated joint kinematic, muscle forces, joint torques, and JRs on the MSK modeling choices (Martelli et al., 2015; Myers et al., 2015; Roelker et al., 2017; Zuk et al., 2018). Zuk et al. (2018) used the generic gait2392 (Delp et al., 1990) and gait2354 (Anderson and Pandy, 1999) models, both available in OpenSim (Delp et al., 2007), to investigate the effect of the model input perturbation on the magnitude and profile of the muscle forces. They analyzed factors such as the maximum isometric force, segment masses, location of the hip joint center, number of muscles, and use of different dynamic simulation methods. Myers et al. (2015) developed an open-source probabilistic MSK modeling framework to assess how measurement error and parameter uncertainty such as marker placement, movement artifacts, body segment parameters, and muscle parameters propagate through a gait simulation. They used the gait2392 OpenSim model (Delp et al., 1990) and concluded that the effect of the parameter changes, resulting in mean bounds that ranged from 2.7 to 8.1 Nm in joint moments, 2.7°-6.4° in joint kinematics, and 35.8 to 130.8 N in muscle forces. The effect of the different joint axis definitions has been studied by Martelli et al. (2015) that found an average variation of 2.38° and 0.33 of body weight (BW) for the joint angles and joint forces, respectively. Roelker et al. (2017) made a global comparison of joint kinematics, muscle activation and force during gait between four different OpenSim models (Delp et al., 1990; Arnold et al., 2010; Hamner et al., 2010; Caruthers et al., 2016). Their study showed that differences in coordinate system definition and muscle parameters may significantly impact the simulation results. In addition, they found that among all factors, muscle parameters, skeletal anatomy, coordinate system definition, virtual marker location, and scale factors influence kinematics and kinetics outputs the most.

To the knowledge of the authors, there are no studies in the literature that identify and quantify the uncertainties related to the boundary conditions, due to differences in the definition of generic MSK models, which are used as inputs to coupled finite element models. This study aims to investigate this aspect by comparing predicted TKR kinematics and knee joint forces during level walking obtained from three different generic MSK models, each scaled to fit the patient of the Fifth Knee Grand Challenge (KGC) (Fregly et al., 2012).

## Materials and Methods

### Experimental Data

Experimental data obtained from the fifth edition of the KGC Competitions (Fregly et al., 2012; Kinney et al., 2013) of a specific patient labeled PS (age: 86, height: 180 cm, and mass: 75 kg) were used in this study. In particular, a standing reference trial (*PS_staticfor2*) and four overground gait trials at the self-selected speed of the subject (*PS_ngait_og*, trials *1, 7, 8*, and *11*) were used for the model scaling and walking simulations, respectively. The *in vivo* forces and moments acting on the left knee joint were available from six load cell sensors embedded in the tibial implant (eTibia). Motion capture data were synchronized and preprocessed using an *ad hoc* MATLAB® script in order to select the time interval for the gait cycle (i.e., between two consecutive heel strikes of the left foot) and to obtain OpenSim input files. The ground reaction force data, computed about the center of pressure, were filtered with a zero-lag fourth-order low-pass Butterworth filter with a cut-off frequency of 30 Hz.

### Musculoskeletal Models

**Figure 1:**
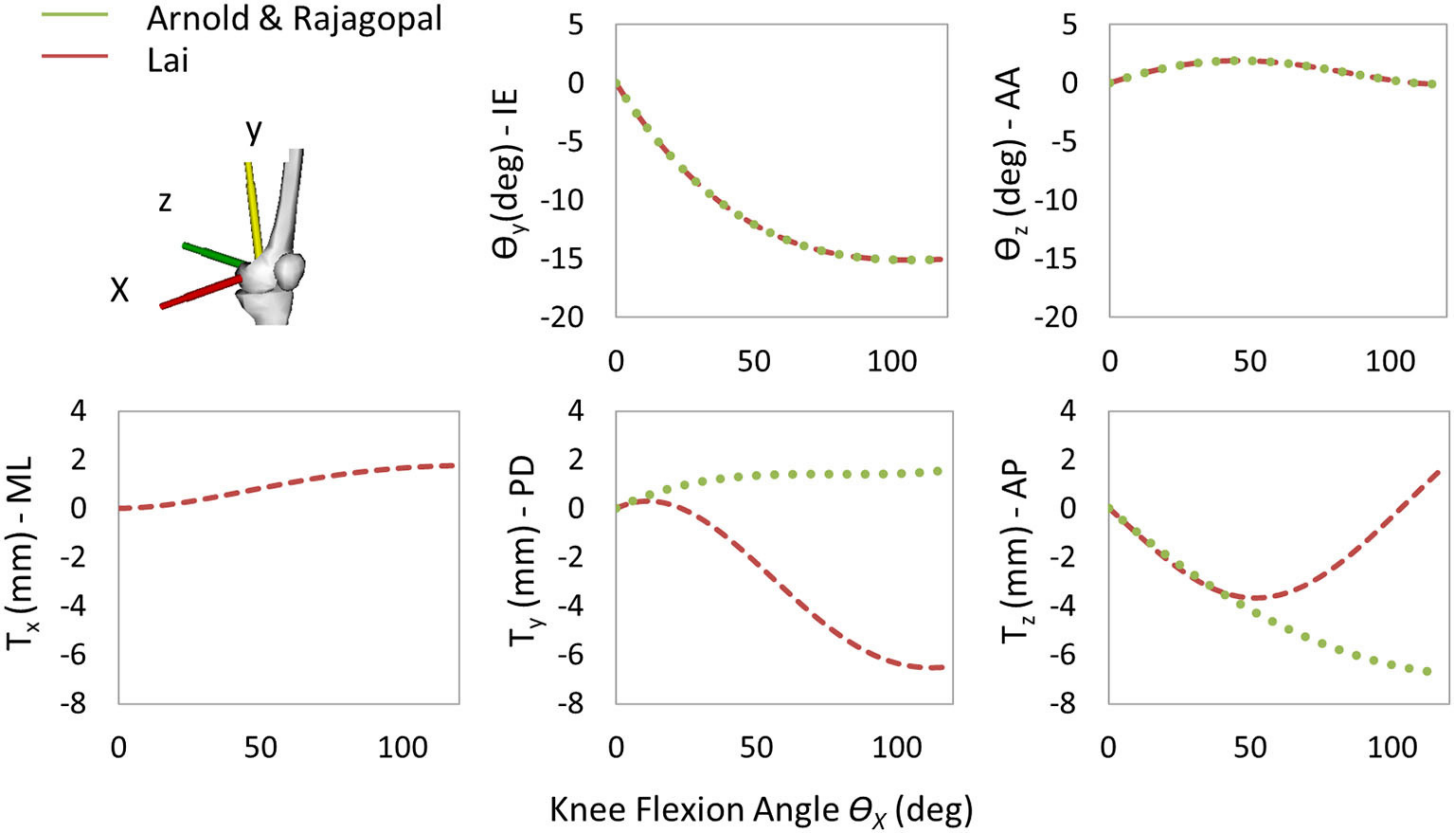
Trends of the coupled translations and rotations of the tibia with respect to the femur as a function of the knee flexion angle for the three models. Only one curve is reported for Arnold and Rajagopal since the same knee spline functions are implemented in the models.

The following three generic MSK models were selected for this study: Lower Limb Model 2010 (Arnold et al., 2010), Rajagopal2016 Full-Body Model (Rajagopal et al., 2016), and Lai2017 Full Upper and Lower Body Model (Lai et al., 2017). The three models, called hereinafter Arnold, Rajagopal, and Lai, respectively, are available on the OpenSim's website and shared the same body segment geometries, inertial properties, and body coordinate reference systems (CRS). The inertial properties come from a cohesive set of 21 cadaveric specimens (height: 168.4 ± 9.3 cm and mass: 82.7 ± 15.2 kg) (Ward et al., 2009), and the bone geometries were created by digitalizing a set of bones from a 170-cm tall male subject. While anatomically similar, these three models implement different tibiofemoral joint (TFJ) kinematics and muscle models. Arnold is based on the gait2392 lower limb model (Delp et al., 1990) and uses the equations reported in the study by Walker et al. (1988) for the knee joint kinematic definition. Compared with gait2392, the Arnold model more accurately describes the muscle geometries and physiology. Rajagopal tries to overcome some of the major limitations of the Arnold model (i.e., extensive use of ellipsoidal wrapping surfaces and muscle parameters suited for elderly individuals). Finally, the Lai model is an improved version of the Rajagopal model capable of simulating pedaling and fast running in addition to walking. The TFJ kinematics is slightly modified in Lai compared with the Arnold and the Rajagopal model where the same equations are used to define anterior–posterior (AP) and proximal–distal translation, internal–external (IE), and abduction–adduction (AA) rotation as function of the flexion–extension (FE) degree of freedom. It is important to notice that in the Lai model, the medial–lateral (ML) translation was also included in the TFJ kinematics, and an offset of 3.6, −1.7, and 1 mm in the X-, Y-, and Z-direction, respectively, was introduced to reposition the tibia with respect to the femur. Main differences among the three models are reported in Table 1. A comparison of the trends of the coupled translations and rotations of the tibia with respect to the femur as a function of FE angle is shown in Figure 1.

**Table 1:** Main differences among the three models, i.e., Arnold, Rajagopal, and Lai.

**Model**	**Arnold**	**Rajagopal**	**Lai**
Muscle model	Schutte (Schutte et al., 1993) + Thelen (Thelen, 2003)	Millard (Millard et al., 2013)	Millard (Millard et al., 2013)
Muscle parameters	Ward (Ward et al., 2009)	Ward (Ward et al., 2009) + Handsfield (Handsfield et al., 2014)^a^	Ward (Ward et al., 2009) + Handsfield (Handsfield et al., 2014)^b^
Kinematic of the TFJ	Walker (Walker et al., 1988)	Walker (Walker et al., 1988)	Modified Walker (Walker et al., 1988)
Range of motion for FE rotation	[0–100°]	[0–120°]	[0–140°]
Coordinates implemented in the TFJ^c^	FE, IE, AA rotations PD, AP translations	FE, IE, AA rotations PD, AP translations	FE, IE, AA rotations ML, PD, AP translations

a *Lower extremity muscle architecture was improved by combining the cadever-based estimates of optimal muscle fiber lengths and pennation angles derived by Ward et al. (2009) with magnetic resonance imaging (MRI) muscle volume data of 24 young healthy subjects reported in Handsfield et al. (2014)*.

b *The muscle tendon parameters and paths defined in the Rajagopal model were updated for 22 muscle tendon units (11 per leg). They did not change the muscle maximum isometric forces*.

c *The FE is the only indipendent coordinate implemented in the TFJ for all the three models*.

**Figure 2:**
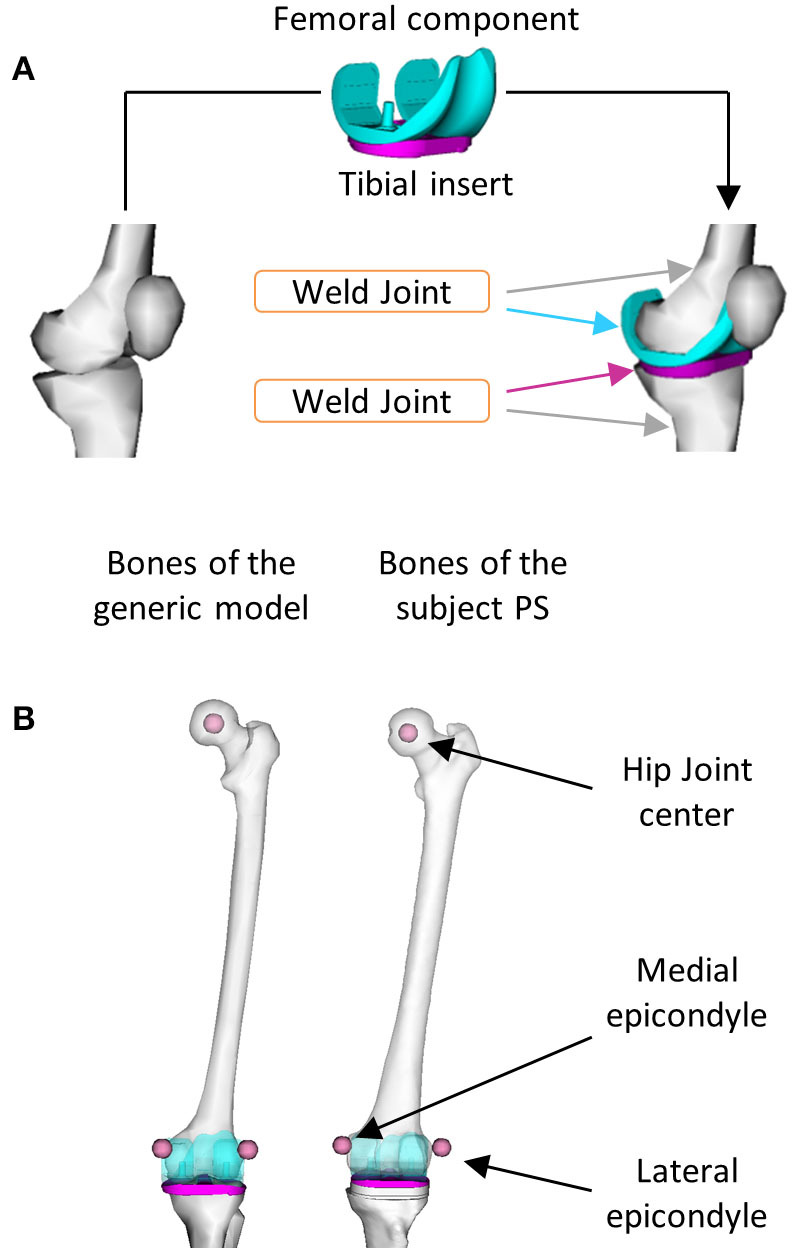
Procedures used to include the implant components in the model: **(A)** Two welding joints were defined to connect the femoral component to the left femur and the tibial insert to the left tibia and **(B)** Bony landmarks were identified in the left femur of the generic model and in the one of the subject PS.

The generic models were scaled to approximate the anthropometry of the subject under this study. Then, the femoral component (FC) and the TI geometries were added to the kinematic chain to enable the study of the implant kinematics, relevant for any FEA application. In particular, the FC was rigidly connected to the left femur and the left tibia to TI using two welding joints (Figure 2A). The geometry. *stl* files of the implant were converted in. *vtp* format files by using the nmsBuilder software^1^ (Valente et al., 2017). To find the relative position and orientation between the implant components and the corresponding bones, the provided *Full Leg.wrp* geometry file was used as a reference. This file includes bone geometries of the left leg of the subject with properly positioned and oriented implant components. The realistic alignment was thus reproduced by identifying bony landmark positions (i.e., hip joint center and medial and lateral epicondyles) in the femur of the generic model and one of the subject PS as shown in Figure 2B. The same virtual marker set (47 markers) defined by looking at the experimental marker positions was assigned to all the three models.

### Simulation Workflow

**Figure 3:**
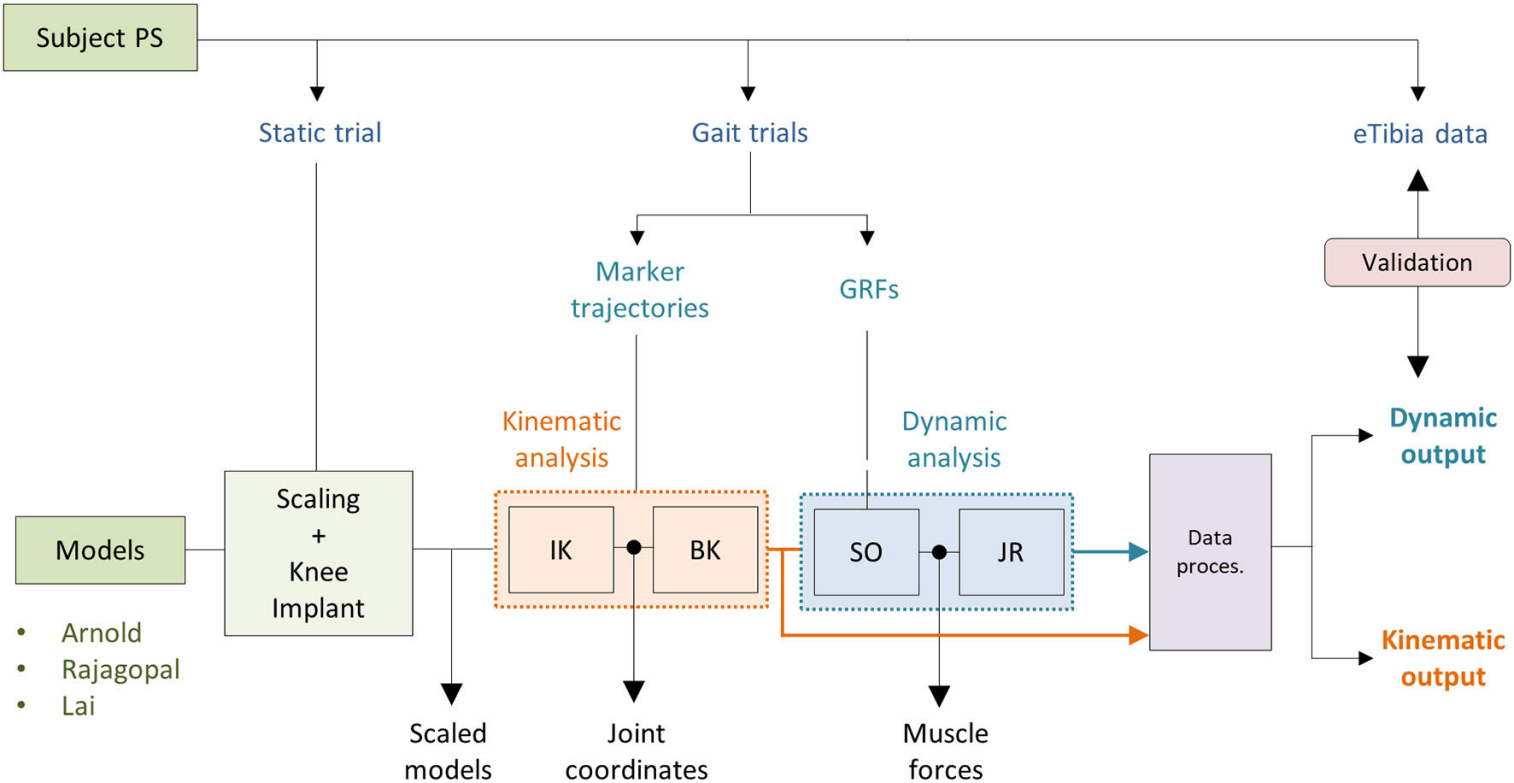
Simulation workflow performed using OpenSim 3.3 and Matlab®.

The simulations were performed using OpenSim 3.3 and MATLAB®. As shown in Figure 3, two main analyses were conducted as follows: a kinematics analysis to estimate the relative pose of the TI with respect to the FC (kinematic output) and a dynamic analysis to compute the loads acting between the two implant components (dynamic output). For all three models, the same setup files used as inputs to the simulations were employed.

#### Kinematic Analysis

Joint coordinates were first computed using the inverse kinematic (IK) OpenSim tool that solves a weighted least square problem to best reproduce the experimental kinematic data by minimizing the distance between corresponding pairs of virtual and experimental markers (Lu and O'Connor, 1999). The body kinematic (BK) tool was then used to obtain information about the pose of the TI and the FC during the simulated gait task (Figure 3). BK records the orientation and the position of the reference system of each body (located at the center of mass) with respect to the ground CRS.

#### Dynamic Analysis

Muscle forces were first estimated using the static optimization (SO) approach. SO solves the muscle redundancy problem by minimizing the sum of muscle activations squared (Anderson and Pandy, 2001). Ideal force generators (i.e., reserve actuators) to ensure dynamic consistency were added about each joint. A unitary maximum force was assigned to prevent them from altering the muscle recruitment by taking too much of the joint torque. Residual actuators with a maximum generalized force of 100 N were applied to the pelvis joint. Also, the maximum isometric force of all the muscles involved in the FE of the left knee was reduced by 35%. A reduced strength of the flexor/extensor muscles has been reported for patients who undergo TKR (Marra et al., 2015). The JR analysis (Steele et al., 2012) was then performed to compute the knee JR forces and moments acting on the tibia (Figure 3).

#### Comparison of Results and Model Validation

The data obtained from the kinematic and dynamic analyses were processed with an *ad hoc* Matlab® script (Figure 3). In this step, the pose of the TI relative to the FC during the simulated gait task was computed. The forces and moments acting on the tibia, estimated using the JR analysis, were first referred to the TI coordinate reference system and then transformed in order to compare them with the measured loads of the instrumented eTibia device. The coordinate system of the eTibia load measurements was, in fact, not consistent with the body CRS of the TI. Medial and lateral contact forces (*F*_*MED*_ and *F*_*LAT*_) were computed using the regression equations provided by the KGC competition dataset that consider the terms related to the superior–inferior force (*Fz*_*T*_) and varus–valgus moment (*My*_*T*_):

(1) $$\begin{array}{r} F_{MED}\;=\;0.510\;Fz_{T}\;+\;0.0213\;My_{T} \end{array}$$

(2) $$\begin{array}{r} F_{LAT}\;=\;0.49\;Fz_{T}\text{-}0.0213\;My_{T} \end{array}$$

where the force is expressed in N and the moment in N mm. The total compressive contact force (*F*_*TOT*_) is then computed as the *F*_*LAT*_ + *F*_*MED*_.

Based on the four gait trials, mean and SD were computed for both predicted and measured quantities in each time interval of the gait cycle. Also, maximum SD and maximum variation range (VR) metrics were calculated to quantify within and between model prediction variability, respectively. The first index is a measure of dispersion defined as the maximum value of the SD computed at each time interval and considering the simulation results of all the four trials obtained with the same model. The maximum VR is a measure of variability between model predictions obtained considering the maximum difference between the minimum and maximum mean value calculated at each time interval with the three models. In order to validate the simulation results and quantify the difference between model prediction and experimental measurements obtained from the instrumented implant, root mean squared errors (RMSE) and the coefficient of determination (*R*^2^) were computed individually for each trial and hereby reported as mean across the four trials.

## Results

### Kinematic Results

**Figure 4:**
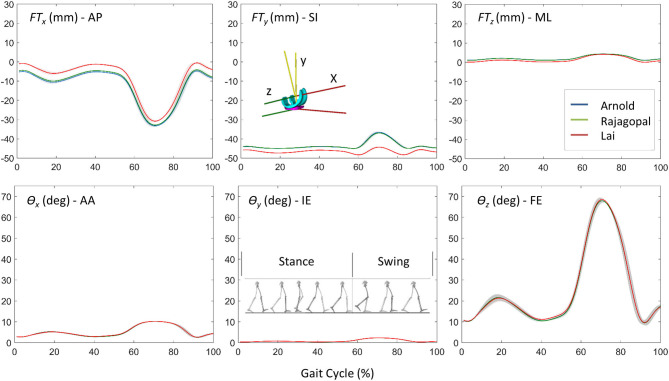
The comparison of kinematic results for the three models: position vector components (*FT*_*x*_, *FT*_*y*_, and *FT*_*z*_) and Euler angles (θ*x*, θ*y*, and θ*z*) that defined the pose of the TI with respect to the FC during the simulated gait trial.

The Euler angle (θ_*x*_, θ_*y*_, and θ_*z*_) trajectories and the position vector components (*FT*_*x*_, *FT*_*y*_, and *FT*_*z*_) that defined the pose of the TI with respect to the FC during the simulated gait trial are shown in Figure 4. The curves had similar trends but non-negligible differences in magnitude were observed especially in the AP and SI translations. The VR was lower than 0.9° for the three Euler angles and 1 mm for the ML translation, while maximum VR values equal to 4.65 mm and 7.72 mm were observed during the stance phase in the AP translation and during the swing phase for the SI translation, respectively (Table 2). Within and between model variability in terms of maximum SD values and VR are reported in Table 2 for all the six quantities that define the kinematic output.

**Table 2:** Within and between model variability in terms of maximum SD values and variation range for all the quantities that define the kinematic and dynamic musculoskeletal simulation output.

	**Kinematic output**						**Dynamic output**					
		**FTx (mm)**	**FTy (mm)**	**FTz (mm)**	****θ**x (deg)**	****θ**y (deg)**	****θ**z (deg)**	**Fx (N)**	**Fy (N)**	**Fz (N)**	**Mx (Nm)**	**My (Nm)**	**Mz (Nm)**
Max SD	Arnold	2.87	1.18	0.39	0.22	0.97	5.57	124.3	293.7	15.54	6.87	3.29	13.5
	Rajagopal	2.88	1.15	0.41	0.22	0.98	5.59	93.37	228.61	20.04	5.07	3.09	3.14
	Lai	2.92	0.72	0.48	0.22	0.98	5.62	97.33	235.8	17.67	4.94	2.82	3.57
Max VR		4.65	7.72	1.03	0.032	0.194	0.84	222	933.61	112.53	7.76	2.91	40.85

### Dynamic Results and Model Validation

**Figure 5:**
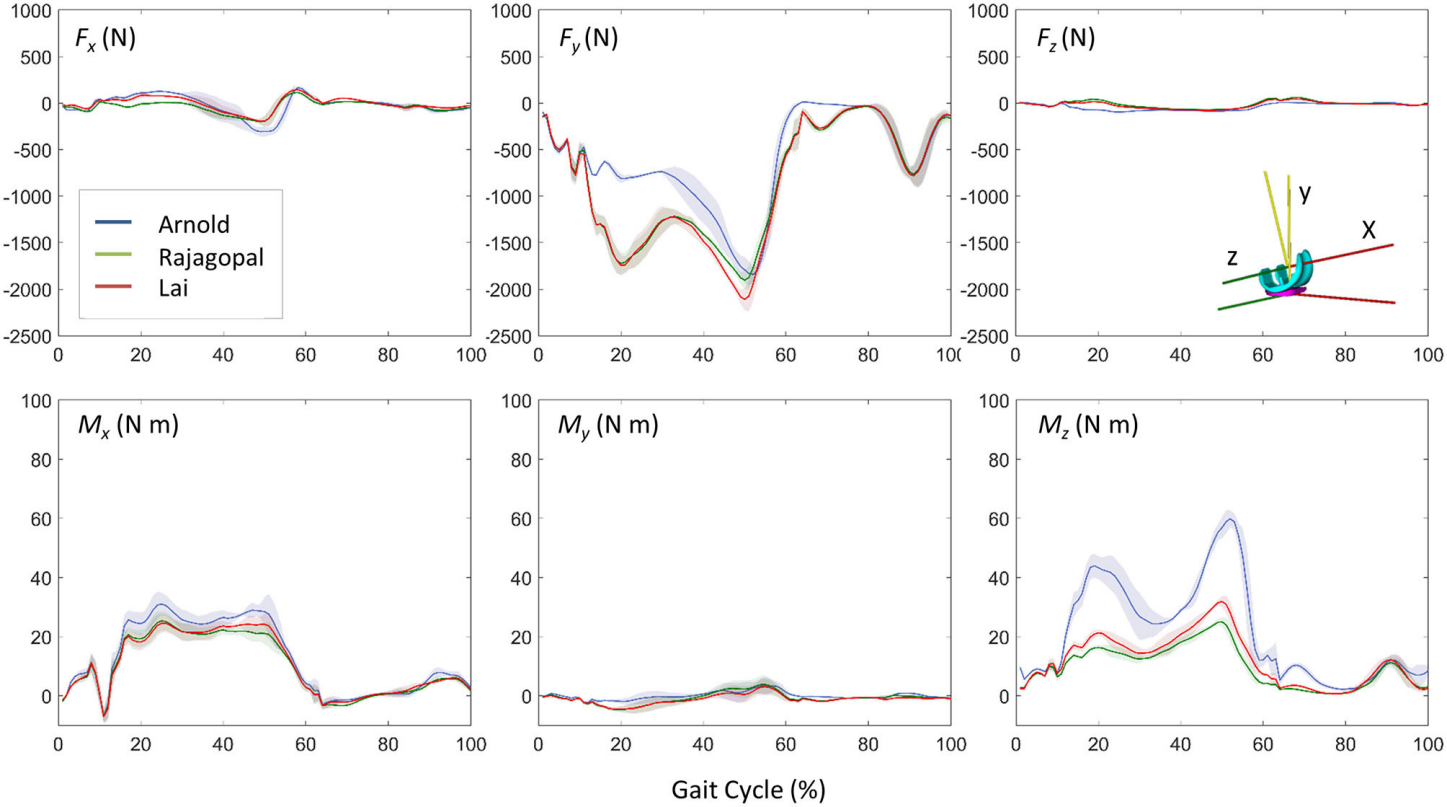
Dynamic results comparison for the three models: three force and moment components acting on the TI predicted by the musculoskeletal models (mean ± SD).

**Figure 6:**
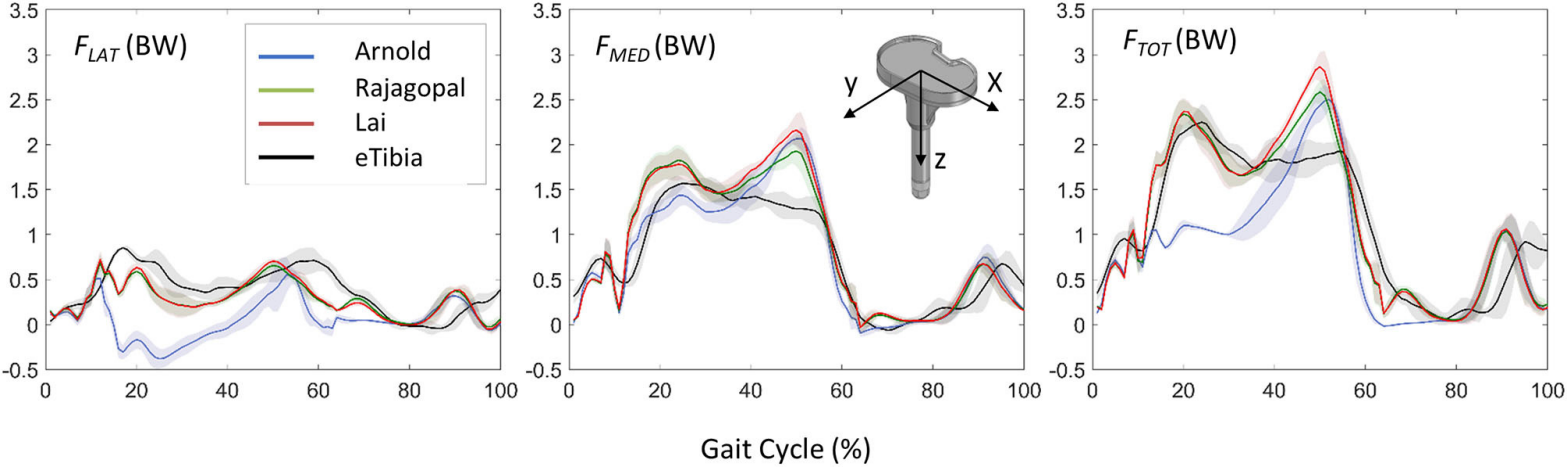
Comparison between the predicted loads and the *in vivo* measurements in terms of lateral, medial, and total forces acting on the TI component and expressed in the coordinate reference systems of the instrumented eTibia device.

Figure 5 shows the JR forces and moments acting on the TI and expressed in the CRS of the TI component, as predicted by the dynamic MSK simulations. Maximum SD and VR values were found large for the normal force and the moment about the FE axis (Table 2). Maximum VR values of about 930 N and 40 Nm were observed for *Fy* and *Mz*, respectively, in correspondence of the first characteristic peak (i.e., ~20% gait cycle, during heel strike). Medial, lateral, and total knee joint contact forces acting on the TI as predicted by the three MSK models and measured *in vivo* via the instrumented implant are shown in Figure 6. Two typical force peaks at approximately the time of the toe-off and heel strike can be observed in the medial, lateral, and total contact forces. Experimental data from the instrumented knee implant reported maximum values of the total knee contact force of about 2.2 BW and 1.9 BW for the first and second peak, respectively, while predicted values ranged from about 1 BW to 2.3 BW and from 2.4 BW to 2.9 BW. Lateral forces were in general underestimated by all the three models while the medial forces were slightly overpredicted. Results in terms of RMSE and *R*^2^ for the total contact force are reported in Table 3 together with the maximum SD for the measured and predicted medial, lateral, and total contact forces. The mean RMSE of total contact force across the models range from 0.38 and 0.62 BW and *R*^2^ values range from 0.22 to 0.80.

**Table 3:** Maximum SD for the predicted and measured *F*_TOT_, *F*_LAT_, and *F*_MED_.

	**Max SD**			**RMSE F_**TOT**_ (BW)**	***R*^**2**^ F_**TOT**_**
	**F_**LAT**_ (BW)**	**F_**MED**_ (BW)**	**F_**TOT**_ (BW)**		
Arnold	0.28	0.23	0.4	0.62	0.22
Rajagopal	0.14	0.22	0.31	0.38	0.80
Lai	0.16	0.25	0.32	0.43	0.77
eTibia	0.18	0.25	0.37	–	–

*Root mean squared error (RMSE) and coefficient of determination (R^2^) are computed for the results of three models considering the total contact force acting at the tibial insert component*.

## Discussion

The aim of this study was to compare the simulation results obtained from three different generic MSK models, scaled to fit the patient of the Fifth KGC, in terms of TKR kinematics and knee joint force during level walking which can be used as boundary conditions in a coupled finite element model when scaled to fit the patient of the Fifth KGC (Fregly et al., 2012). The estimated knee JRs were also compared with those measured by the instrumented knee implant to quantify the “global goodness of fit” for each model. As we did not have a true value for the knee kinematics (typically provided by fluoroscopy, not available in the Fifth KGC for the gait trials analyzed in this study), we could only compare these in terms of relative differences between the three models. In the predicted relative pose of the two implant components, for example, a slight shift in the AP and SI translation curves was found for the Lai model. This can be justified by the different tibia positions with respect to the femoral body as described in the “Musculoskeletal Models” section and by the different kinematic definitions of the knee joint as explained in Figure 1. The most recent Lai model was developed to better predict the activation pattern of muscles during walking, pedaling, and fast running. It considers an increased range for the flexion angle and a different knee spline function definition for the custom joint. Small differences can be observed by looking at the kinematic results suggested by the Rajagopal and Arnold models (<12.6% maximum percentage difference for AP, SI, and ML translations and <1% for AA, IE, and FE rotations). This is probably due to a non-identical definition of the whole kinematic chain which might result in a non-identical solution of the scaling and IK problem. Rajagopal and Arnold used the same spline functions for the TFJ, but differences can be observed, for example, in the knee FE range or the patellofemoral joint definition.

A comparison of the results obtained in terms of knee implant kinematics with respect to the literature is complicated by the following two facts: on the one hand, only few studies are focused on the use of MSK models to predict the relative pose of the knee implant components during gait, and on the other hand, in these studies, different coordinate systems are used to monitor the relative pose of the two implant components. For instance, in the study proposed by Zhang et al. (2017), which used the same experimental data obtained from the KGC, the FE, IE rotations, and AP translations estimated by the MSK simulations were considered as inputs to the finite element model. The trend of the FE angle is very similar to the one obtained in this study; however, the differences observed by comparing the results related to the other two movements might be due to a different initial location of the origins of the FC and TI CRS.

The accuracy of the three models in predicting the force transmitted at the knee joint was found comparable with that reported in previous studies (Marra et al., 2015; Chen et al., 2016; Andersen, 2018). The correlation between the prediction and measured values of the model is pretty low for the Arnold's model (*R*^2^ = 0.22), better for the Lai's model (*R*^2^ = 0.77), and even better for the Rajagopal's model (*R*^2^ = 0.80). The average error, expressed in terms of RMSE was 0.62, 0.38, and 0.43 BW for the Arnold, Rajagopal, and Lai models, respectively. An overestimation of the total contact force can be observed by looking at the results obtained with all the models in correspondence of the second peak; this might be due to compensatory patterns adopted by the patient. It is not unusual in knee replacement patients to see as compensatory pattern a shift of the load to the other leg during the bipodal phase.

The important differences in terms of predicted joint forces between the three models can be justified by the adoption of a different musculotendinous dynamics model and muscle architectural parameters. Arnold uses the old Schutte1993Muscle model (Schutte et al., 1993) with exceptions on the back muscles that were adapted from the gait_2393 OpenSim model and remain as Thelen2003Muscle format (Thelen, 2003). These muscle models have been updated with the Millard2012EquilibriumMuscle used in Rajagopal and Lai which seems to better describe the specific patient physiology. The characteristic curves defining musculotendon behavior (i.e., tendon force-length, fiber force-length, and force-velocity curves) were improved and more force-generating properties of realistic muscles (e.g., maximum isometric force, optimal fiber length, fiber pennation angles, and tendon slack length) based on magnetic resonance images of healthy subjects were considered (Handsfield et al., 2014). Lai implements further improvements aimed to decrease co-activation of antagonist muscles due to excessing passive force generated by knee and hip extensors.

The adjustments implemented in Lai's model (briefly described in “Musculoskeletal Models” section), which proved to be useful to better predict fast walking and running with respect to Rajagopal's model (Lai et al., 2017), did not produce an improvement in predicting joint forces for a TKR subject. This points out a critical aspect in the use of generic MSK models, also discussed in a previous study (Silva et al., 2021): the need of adapting modeling features in the context of their intended use, for example, exploring and describing common pathology-specific patterns. This overlaps with the theme of personalized models.

Some limitations of this study are important to be mentioned. First, only three generic MSK models were used in the comparative analyses. However, as also mentioned in the “Introduction” section, they were selected because they use the same body geometries, inertial properties, and body coordinate systems. This allows the authors to evaluate the only effect of the different joint kinematic definitions and the different muscle model implementations on the simulation results. Among the factors that mostly affect model predictions are in fact discrepancies in coordinate system definitions, virtual marker sets, and body geometries (Roelker et al., 2017). Another important limitation that is worth mentioning is related to the fact that only gait trials performed by one subject are simulated. Additional analyses that also evaluate inter-subject variability and movement tasks other than level walking should be considered in future studies.

## Conclusion

This comparative study demonstrated that selecting a generic MSK model is an important step. Modelers should be aware of the effect of the MSK modeling assumptions and their influence on the kinematic and dynamic results that depending on the context of use might or might not be critical. This is obviously a delicate point if the results of the MSK simulations are the main outcomes of the analysis but also if they are used as inputs for finite element models in a combined MSK-FEA approach. This study represents a good starting point for future investigations that will evaluate how uncertainties related to MSK models propagate in the FEA results. Detailed studies on uncertainty quantification analyses in the combined MSK-FEA approach will undoubtedly provide important insight in the context of model credibility assessment for the biomechanics community.

## Data Availability Statement

The raw data supporting the conclusions of this article will be made available by the authors, without undue reservation.

## Author Contributions

CC, FDP, and LM: conceptualization and methodology. CC, GD, and LM: simulations and data processing. CC and GD: data curation. CC: writing—original draft preparation and visualization. CC, GD, LM, FDP, and MV: writing—review and editing. FDP and LM: supervision. MV: funding acquisition. All authors contributed to the article and approved the submitted version.

## Conflict of Interest

The authors declare that the research was conducted in the absence of any commercial or financial relationships that could be construed as a potential conflict of interest.

## Publisher's Note

All claims expressed in this article are solely those of the authors and do not necessarily represent those of their affiliated organizations, or those of the publisher, the editors and the reviewers. Any product that may be evaluated in this article, or claim that may be made by its manufacturer, is not guaranteed or endorsed by the publisher.

## Footnotes

^1^ http://www.nmsbuilder.org/
